# Scalable ^68^ Ga Cold Kit Radiolabeling: High-Throughput Preparation of [ ^68^ Ga]Ga-Edotreotide via Aliquoting and Dual Generator Strategy

**DOI:** 10.1055/s-0045-1814733

**Published:** 2026-01-05

**Authors:** Johanne Vanney, Jade Torchio, Stéphane C. Renaud, Marine M. Cadet, Léa Rubira, Cyril Fersing

**Affiliations:** 1 Department of Nuclear Medicine, Institut Régional du Cancer de Montpellier (ICM), Univ. Montpellier, Montpellier, France; 2 IBMM, Univ Montpellier, CNRS, ENSCM, Montpellier, France

**Keywords:** edotreotide, radiolabeling, gallium-68, radiopharmaceuticals, PET imaging, neuroendocrine tumors

## Abstract

**Objectives:**

The aim of the study was to develop and assess alternative radiolabeling strategies for SOMAKIT-TOC using gallium-68, with the aim of improving cost-efficiency by increasing the number of examinations per cold kit while maintaining radiochemical quality suitable for clinical use.

**Materials and Methods:**

Four protocols were evaluated using triplicate test radiolabelings: (1) the reference method using a single generator and a full kit; (2) dual-generator elution into a full kit; (3) single-generator elution into two sequentially radiolabeled aliquots from one kit; and (4) dual-generator elution into two sequentially radiolabeled aliquots from one kit. Radiochemical purity (RCP) was assessed by radio-thin layer chromatography (rTLC) and radio-high performance liquid chromatography (rHPLC), and radiochemical stability was monitored over 4 hours. Additional quality controls parameters included pH, sterility, and peptide integrity in aliquot solution. Procedures 1 and 3 were further assessed in clinical routine.

**Results:**

All protocols yielded radiopharmaceuticals with mean RCP values above 95% by rTLC. Procedure 2 significantly increased the final activity compared with Procedure 1. Procedure 3 enabled doubling the number of examinations per kit while maintaining RCP comparable to the reference method Procedure 4 showed greater variability. Radiolabeled products remained stable over 4 hours as assessed by rTLC, although rHPLC revealed a gradual decrease in purity. In clinical use, Procedure 3 allowed 94 preparations that met diagnostic needs, though 33% had RCP between 80 and 95%.

**Conclusion:**

Aliquoting a single cold kit for dual radiolabeling using one generator is feasible and cost-effective, although it is associated with greater RCP variability. Dual-generator strategies yield higher activity but may further compromise RCP. Further optimization is required to improve robustness of off-label protocols while preserving the highest product quality.

## Introduction

**Fig. 1:**
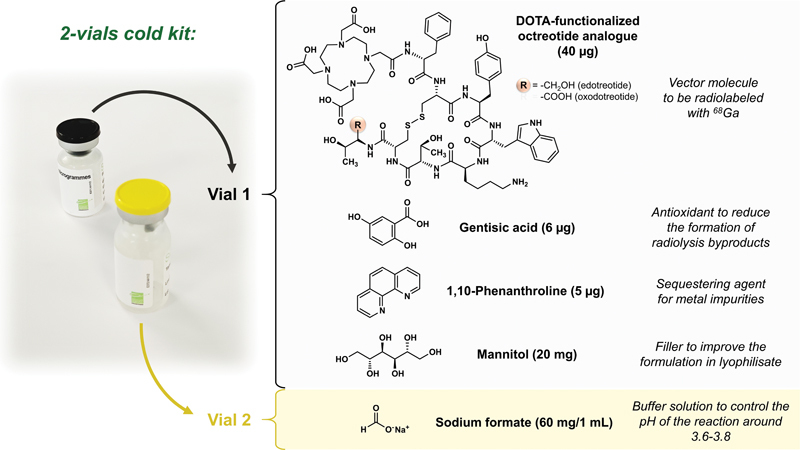
Contents of commercially available cold kits of DOTA-conjugated SSTr ligand for ^68^ Ga radiolabeling (either NETSPOT or SOMAKIT-TOC). SSTr, somatostatin receptor.

The formulation of a molecular imaging vector as a cold kit for radiopharmaceutical preparation is a well-established approach in nuclear medicine and is widely applied to SPECT imaging agents labeled with technetium-99m. 1 Over the past decade, kit-based radiolabeling with gallium-68 ( ^68^ Ga) has also gained traction, a pathway first pioneered by DOTA-functionalized somatostatin analogs. 2 Notably, DOTATATE (NETSPOT, Novartis) and DOTATOC (SOMAKIT-TOC, Novartis) were both approved in 2016 and have since been extensively used for PET imaging of somatostatin receptor (SSTr) overexpression, 3 4 enabling localization of primary tumors and metastases in adult patients with gastroenteropancreatic neuroendocrine tumors and other SSTr-positive cancers. 5 These cold kits, provided under inert atmosphere in lyophilized form, contain an excess of the vector molecule to be radiolabeled, along with the necessary excipients to ensure efficient and reproducible radiolabeling ( Fig. 1 ). 6 These typically include a buffer (e.g., sodium formate), an antioxidant (e.g., gentisic acid), a co-ligand (e.g., 1,10-phenanthroline) to mitigate the impact of trace metal contaminants, and a filler (e.g., mannitol), which may also contribute to the stability of the final radiopharmaceutical. 7 8

Radiopharmaceutical preparation using ^68^ Ga remains a relatively expensive activity. 9 In Europe, the cost of a ^68^ Ge/ ^68^ Ga generator amounts to several tens of thousands of euros, and its progressive decay necessitates replacement every 6 to 11 months. In addition to the costs of routine supplies and initial investments (high-energy shielded cell, small shielded equipment suitable for ^68^ Ga), the price of commercially available ^68^ Ga cold kits is currently quite prohibitive. Consequently, cost-effectiveness is a major concern for hospital-based radiopharmacy units, particularly in view of the implementation of this activity. In this context, we explored several off-label strategies to optimize the cost-efficiency of ^68^ Ga radiolabeling of octreotide analogs formulated as cold kits. Specifically, the use of two generators to label a single kit was investigated, with or without splitting the kit contents into two aliquots for separate radiolabeling procedures.

## Methods

### General Information

Radiolabeling experiments were conducted using commercial SOMAKIT-TOC (edotreotide) kits. All supplies and materials used during these experiments were identical to those employed in routine clinical practice. Gallium-68 was obtained by eluting pharmaceutical-grade ^68^ Ge/ ^68^ Ga generators (GALLIAD 1850 MBq, IRE Elit, Belgium; age of generator 1:1 month post-calibration; age of generator 2:10 months post-calibration) with approximately 1.1 mL of 0.1 M hydrochloric acid each. For experiments involving a single generator, only generator 1 was used. Radiopharmaceutical production was performed within a dedicated radiopharmacy unit, in a GMP grade C (ISO 7) cleanroom, using a shielded GMP grade A (ISO 5) isolator equipped with laminar airflow (MEDI 9000 Research 4R, LemerPax, France), where the ^68^ Ge/ ^68^ Ga generators were installed. Heating of the kit vial to allow radiolabeling was performed using the heating block of a LUNA module (Elysia-Raytest, Germany), controlled via the dedicated software (GAIA Control, Elysia-Raytest, Germany). Radioactivity in the final product vial and in patient doses was measured using a calibrated ionization chamber (CRC-25R, Capintec, United States).

### Radiolabeling Protocols

**Fig. 2:**
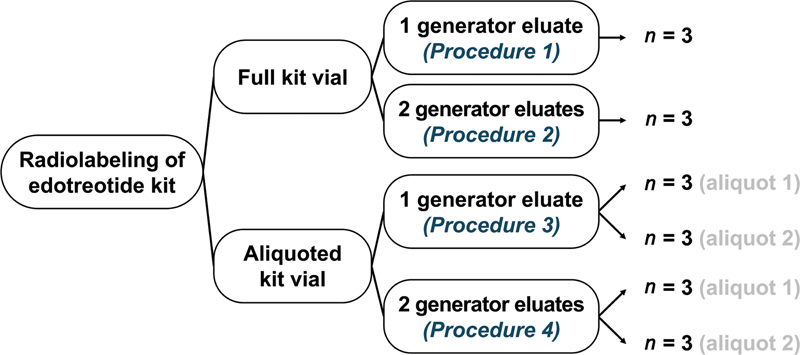
Experimental plan for the study of alternative ^68^ Ga radiolabeling protocols applied to SOMAKIT-TOC.

Alternative protocols to the ^68^ Ga radiolabeling methodology described in the summary of product characteristics (SmPC) of SOMAKIT-TOC were evaluated. These protocols involved the use of either one or two ^68^ Ge/ ^68^ Ga generators for a single radiolabeling procedure. Each radiolabeling was performed either using a full kit vial or a kit vial subdivided into two aliquots. For experiments involving aliquoted kits, radiolabeling runs of the two aliquots were conducted on the same day, with a time interval of 4 to 6 hours between them. Overall, four distinct procedures were investigated: (1) one generator eluate for one full kit vial (reference conditions); (2) two generator eluates for one full kit vial; (3) one generator eluate for one aliquoted kit vial; (4) two generator eluates for one aliquoted kit vial. The different alternative radiolabeling protocols evaluated are summarized in Fig. 2 . Three test radiolabeling runs were performed for each of the four protocols investigated.

For Procedure 1, a full kit vial was reconstituted with 3.1 mL of water for injection (WFI; Eau pour préparations injectables 10 mL PROAMP, Aguettant, France), drawn using a two-part 10 mL syringe (Injekt Luer Solo, B. Braun, Germany) fitted with a coated needle (Sterican 21G 0.80 × 25 mm, B. Braun, Germany). Next, 0.1 mL of the buffer solution supplied in the kit was measured using a low dead volume 1 mL syringe (Injekt-F 1 mL, B. Braun, Germany), diluted to a total volume of 1 mL with WFI, and added to the kit vial. A single ^68^ Ge/ ^68^ Ga generator was then connected to the kit vial using a disposable extension line (Lectrocath 50 cm, Vygon, France), a male–male adapter (male-2-male LL, Vygon, France), and a coated needle. The generator was eluted directly into the kit vial by creating a vacuum using the peristaltic pump of a GAIA module (Elysia-Raytest, Germany). The vial was subsequently heated at 97°C for 8 minutes, then allowed to cool for 5 minutes before measuring its radioactivity at the end of synthesis (EoS). A sample of the final radiopharmaceutical was then collected for quality control testing.

For Procedure 2, a full kit vial was reconstituted with 2 mL of WFI. Then, 0.2 mL of the buffer solution supplied in the kit was withdrawn, diluted to a total volume of 1 mL with WFI, and added to the reaction vial. During the radiolabeling step, two ^68^ Ge/ ^68^ Ga generators were connected to the kit vial and eluted sequentially, directly into the vial. The remaining steps were identical to those described in Procedure 1.

For Procedure 3, a kit vial was reconstituted with 6.2 mL of WFI. Using the same syringe, 3.1 mL was immediately withdrawn from the kit vial and transferred into a sterile type 1 glass vial (TC-ELU-5, Curium, France) to obtain two equal aliquots. The first radiolabeling was performed immediately on one aliquot, following the steps described in Procedure 1. The second aliquot was stored at 4 to 8°C. Between 4 and 6 hours after the first radiolabeling, a second radiolabeling was performed on the remaining aliquot, again following Procedure 1.

For Procedure 4, the kit vial was reconstituted and divided into two aliquots as described in Procedure 3. Both aliquots were then radiolabeled on the same day, 4 to 6 hours apart, using the two-generator preparation method detailed in Procedure 2.

### Radiochemical Purity Assessment

Radiochemical purity (RCP) of [ ^68^ Ga]Ga-edotreotide was determined by radio-thin layer chromatography (rTLC) and radio-high performance liquid chromatography (rHPLC).

For rTLC analyses, two iTLC-SG strips (2 cm × 10 cm) were used. A small drop of [ ^68^ Ga]Ga-edotreotide was applied to each strip, 1 cm above the baseline. The first strip was developed in a chamber containing a mobile phase of 1 M aqueous ammonium acetate/methanol (1:1, v/v) (Condition A), while the second strip was developed in 0.1 M aqueous sodium citrate at pH 5 (Condition B). In both cases, the mobile phase was allowed to migrate until reaching approximately 1 cm from the top edge of the strip. Following development, the strips were removed from the chambers and analyzed using a TLC scanner (miniGITA Star, Elysia-Raytest, Germany). The distribution of radioactivity along the strips was quantified using the corresponding acquisition software (TLC Control v2.30, Raytest, Germany) and data analysis software (GINA Star TLC v6.0, Elysia-Raytest, Germany). According to the SmPC, the retention factor (Rf) values under Condition A were as follows: ^68^ Ga colloids, Rf = 0.0 to 0.2; [ ^68^ Ga]Ga-edotreotide and free ^68^ Ga ^3+^ , Rf = 0.8 to 1.0. Under Condition B, free ^68^ Ga ^3+^ migrated with Rf = 0.8 to 1.0, while [ ^68^ Ga]Ga-edotreotide and ^68^ Ga colloids remained at the origin with Rf = 0.0 to 0.3.

Radio- and UV-HPLC analyses were performed using a Nexera X3 system (Shimadzu, Japan) with HPLC-grade solvents. The chromatographic separation was achieved using a C _18_ ACE Equivalence™ column (3.0 × 150 mm, 110 Å pore size, 3 µm particle size) thermostated at 30°C. The mobile phase consisted of solvent A (0.1% TFA in water) and solvent B (0.1% TFA in acetonitrile), delivered at a constant flow rate of 0.5 mL/min. A segmented gradient elution was applied as follows: 0 to 1 minute: 95/5 A/B; 1 to 11 minutes: linear gradient from 95/5 to 10/90 A/B; 11 to 15 minutes: linear gradient from 10/90 to 95/5 A/B; 15 to 16 minutes: 95/5 A/B (re-equilibration). Data acquisition and processing were performed using the dedicated software package (Gina Star 10, Elysia-Raytest, Germany).

### Radiocomplex Stability Over Time

The RCP of each radiolabeled product was evaluated by both rTLC and rHPLC immediately at EoS and subsequently at hourly intervals for up to 4 hours post-preparation. During this period, the final product was stored at room temperature, maintained at 22 ± 2°C.

### Other Quality Controls on the Radiolabeling Product

Upon completion of synthesis, visual inspection of the [ ^68^ Ga]Ga-edotreotide preparations was performed to confirm the absence of particulate matter and to verify that the product was a clear, colorless solution. Additionally, pH was assessed using MQuant indicator strips (range 2.5–4.5; VWR, Pennsylvania, United States) to evaluate the final compounded radiopharmaceutical.

Sterility testing of radiolabeled products was performed by supplementing a tryptic soy broth tube (CASO-Bouillon TSB 9 mL, Millipore) with 1 mL of the preparation. The tubes were incubated for 7 days at room temperature, followed by an additional 7 days at 32°C under 5% CO _2_ . Positive and negative controls were incubated in parallel under identical conditions. All tubes were visually inspected daily by the unaided eye, with gentle mixing prior to each observation.

### Edotreotide Stability in Solution

To assess the stability of edotreotide dissolved during aliquoting, four lyophilized kits were reconstituted with 3.1 mL of WFI and stored at 4 to 8°C. Immediately after reconstitution and at 1, 2, 3, 8, 12 and 24 hours, HPLC analysis was performed as previously described, with UV detection at 254 nm. The area under the peak corresponding to edotreotide was monitored over time and a regression analysis confirmed the absence of any relationship between area and time, to validate the stability of the compound. The identity of the edotreotide peak was verified by analyzing a reference sample of edotreotide (MedChem Express, New Jersey, United States) under the same conditions.

### Real-Life Experience of the Single-Generator, Aliquot Protocol

At our institution, Procedure 1 and Procedure 3 are routinely used in clinical practice. The RCP values obtained by rTLC for each clinical preparation prior to batch release were collected, and the mean values obtained with Procedure 1 and Procedure 3 were compared using *z* -test (groups with non-statistically different variances). Similarly, for Procedure 3, the mean RCP values of the first radiolabeled aliquot were compared with those of the second aliquot using Welch *t* -test (groups with statistically different variances). Additional comparisons and appropriate statistical tests were performed on routine RCP values according to various parameters (generator batch and age, final preparation activity, operator) in an attempt to identify predictors of radiolabeling failure.

## Results

A total of 18 test radiolabeling runs were performed: 3 for Procedures 1 and 2, and 6 for Procedures 3 and 4. Table 1 summarizes the mean results obtained for each triplicate.

**Table 1:** Mean activity, RCP, and pH values for each triplicate

Procedure		Mean final activity at EoS (MBq)	Mean RCP at EoS (%)		pH
			rTLC	rHPLC	
**1**		913.0 ± 15.4	98.9 ± 0.7	93.1 ± 1.4	3.6
**2**		1,519.3 ± 128.8	98.9 ± 1.1	91.6 ± 1.0	3.6
**3**	Aliquot 1	971.3 ± 36.6	99.0 ± 0.6	91.1 ± 3.4	3.6
	Aliquot 2	922.3 ± 33.6	97.6 ± 0.9	90.8 ± 2.3	3.6
**4**	Aliquot 1	1,432.3 ± 87.1	95.6 ± 1.4	87.2 ± 3.4	3.6
	Aliquot 2	1,379.7 ± 10.5	97.2 ± 3.4	89.6 ± 3.0	3.6

Abbreviations: EoS, end of synthesis; RCP, radiochemical purity; rHPLC, radio-high performance liquid chromatography; rTLC, radio-thin layer chromatography.

**Fig. 3:**
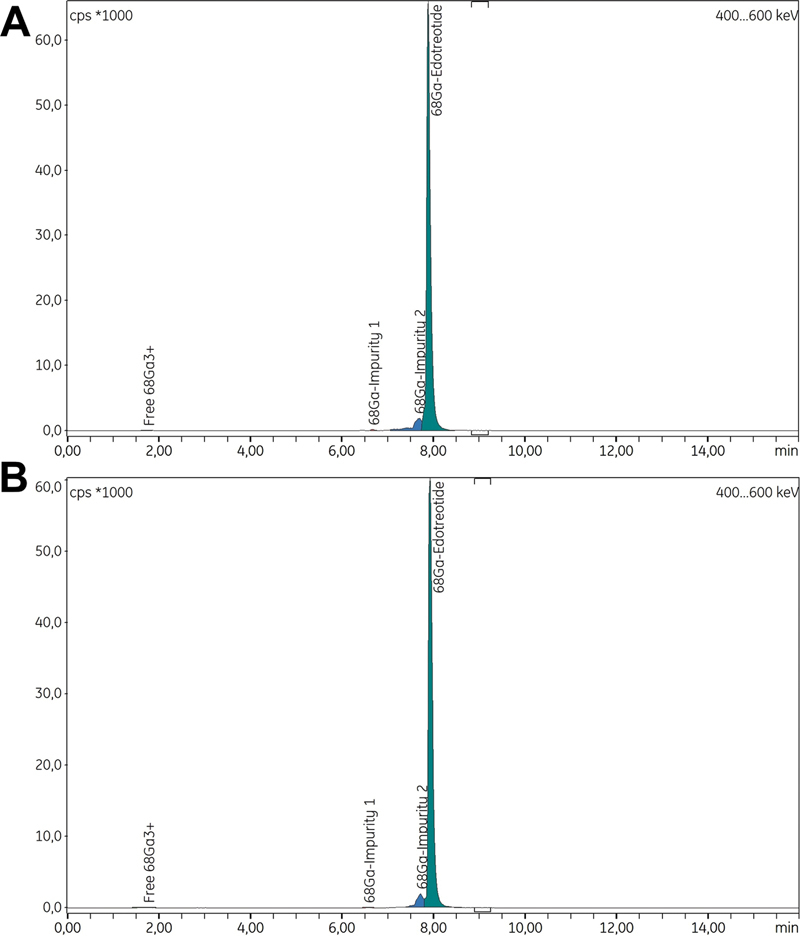
Representative rHPLC spectra of [ ^68^ Ga]Ga-edotreotide, synthesized using either ( **A** ) Procedure 1or ( **B** ) Procedure 3. rHPLC, radio-high performance liquid chromatography.

As expected, preparations performed using Procedure 1 (the method described in the SmPC) yielded radiolabeled products with excellent RCP, with values of 98.9 ± 0.7% by rTLC and 93.1 ± 1.4% by rHPLC. Notably, the RCP values obtained by rHPLC were affected by the presence of a significant byproduct peak eluting just before the [ ^68^ Ga]Ga-edotreotide signal ( Fig. 3A ). Such radiolytic impurities have been previously reported in the literature, including under standard radiolabeling conditions. 10 11 By doubling the amount of buffer solution used in the reaction, Procedure 2, which involves two elutions from GALLIAD generators (i.e., approximately 2.2 mL of ^68^ Ga ^3+^ in HCl 0.1 M), also achieved high RCP (98.9 ± 1.1% by rTLC; 91.6 ± 1.0% by rHPLC). It maintained the pH at 3.6, consistent with the outcome of Procedure 1. Importantly, the mean final activity was significantly increased due to the use of two generators (EoS activity = 1519.3 ± 128.8 MBq vs. 913.0 ± 15.4 MBq with Procedure 1). Using Procedure 3, the RCP remained comparable for both radiolabeling runs, as aliquot 1 yielded 99.0 ± 0.6% by rTLC and 91.1 ± 3.4% by rHPLC. Aliquot 2 yielded 97.6 ± 0.9% by rTLC and 90.8 ± 2.3% by rHPLC. Remarkably, the radiochromatograms obtained from preparations produced with Procedure 3 showed no obvious difference from those resulting from reference radiolabeling, especially in rHPLC ( Fig. 3 ). The mean activities at EoS were slightly lower for aliquot 2 compared with aliquot 1 (922.3 ± 33.6 MBq vs. 971.3 ± 36.6 MBq), due to incomplete recovery of the generator's full elution capacity between the two radiolabeling runs. Finally, radiolabeling of two cold kit aliquots using two generator eluates (Procedure 4) yielded good overall mean RCP values by rTLC, with 95.6 ± 1.4% for aliquot 1 and 97.2 ± 3.4% for aliquot 2. However, one-third of the labeling runs ( *n* = 2/6) resulted in RCP values between 93 and 95%. By rHPLC, the mean RCP values were 87.2 ± 3.4% for aliquot 1 and 89.6 ± 3.0% for aliquot 2. The mean activities at EoS were 1432.3 ± 87.1 MBq and .1379.7 ± 10.5 MBq for aliquots 1 and 2, respectively.

**Fig. 4:**
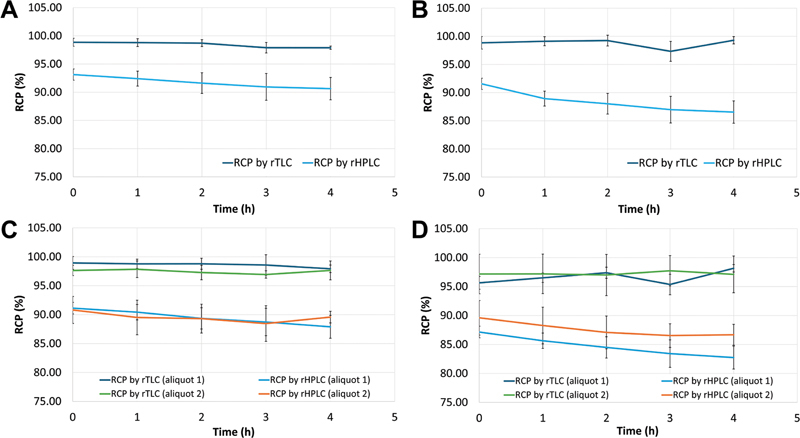
Time course of the mean RCP values, measured in rTLC and rHPLC, of the test radiolabeling products obtained using ( **A** ) Procedure 1, ( **B** ) Procedure 2, ( **C** ) Procedure 3 **,** or ( **D** ) Procedure 4. RCP, radiochemical purity; rHPLC, radio-high performance liquid chromatography; rTLC, radio-thin layer chromatography.

Regardless of the radiolabeling procedure used, the RCP of [ ^68^ Ga]Ga-edotreotide remained highly stable over 4 hours when assessed by rTLC, with mean values at each time point consistently above 95% ( Fig. 4 ). In contrast, RCP values measured by rHPLC showed more pronounced variations over time, likely due to the greater sensitivity of the method, with a decrease of 2 to 5% observed between EoS and 4 hours post-synthesis, depending on the radiolabeling procedure. Specifically, the lowest absolute RCP values observed over time in each condition were 90.4% at *t*_4h_ for Procedure 1, 85.0% at *t*_4h_ for Procedure 2, 85.1% at *t*_3h_ for Procedure 3, and 79.1% at *t*_4h_ for Procedure 4.

Visual inspection and sterility testing of all test radiolabeling products confirmed the production of a clear, colorless, and sterile solution. The pH of all preparations was consistently 3.6. Stability assessment of free edotreotide in aqueous solution demonstrated no change in the area of the corresponding peak in UV-HPLC over 24 hours, when reconstituted aliquots were stored at 4 to 8°C (28.68 ± 1.34 mUA*min at *t*_0_ vs. 30.13 ± 1.26 mUA*min at *t*_24_ , *n* = 4). No statistically significant correlation between the peak area of interest and time could be demonstrated for the four kit samples analyzed (0.31 < *p* < 0.76). The identity of the integrated peak was confirmed by comparing its retention time with that of the edotreotide reference sample, both of which were identical ( *t*_r_ = 7.87min; Supplementary Material ).

In routine clinical practice, a total of 166 preparations of [ ^68^ Ga]Ga-edotreotide using Procedure 1 were performed at our center. The mean RCP of these preparations was 97.3 ± 1.8%, with 0.7 ± 1.0% of free ^68^ Ga ^3+^ and 2.0 ± 1.3% of ^68^ Ga-colloids, as determined by rTLC. Overall, 6.0% of the preparations ( *n* = 10) were non-compliant with the SmPC and displayed a RCP below 95%.

Similarly, 94 preparations of [ ^68^ Ga]Ga-edotreotide were performed in our institution using Procedure 3, corresponding to 47 radiolabeling reactions with aliquot 1 and an equal number with aliquot 2. The overall results showed a mean RCP by rTLC of 94.7 ± 5.1%, with 0.9 ± 1.6% of free ^68^ Ga ^3+^ and 4.4 ± 4.4% of ^68^ Ga-colloids. Notably, the mean RCP values of aliquot 1 and aliquot 2 differed slightly but significantly (93.7 ± 6.0% vs. 95.8 ± 3.8%; *p* = 0.0458). Importantly, 35.1% ( *n* = 33; including 18 aliquot 1 and 15 aliquot 2) of the radiopharmaceuticals prepared using this procedure exhibited an RCP below 95%. The mean RCP obtained with Procedure 3 was significantly lower ( *p* < 0.001) than that obtained with Procedure 1. In this context, predictive factors for radiolabeling failure when using Procedure 3 were investigated. Two different GALLIAD generators were employed during the study period, with 58 radiolabeling reactions performed using Generator 1 and 36 using Generator 2. A statistically significant difference in RCP was observed between preparations (including aliquots 1 and 2 combined), as well as between aliquots 1 and aliquots 2 labeled with either generator (Welch test, *p* < 0.001) ( Table 2 ). For Generator 1, which was used over 11.2 months, a negative correlation was identified between RCP and generator age (Pearson correlation, *p* < 0.001), while only 6.9% (4/58) of preparations from this generator showed an RC *p* < 95%. Conversely, no statistically significant correlation was found for Generator 2 (Pearson correlation, *p* = 0.416), used over 5.2 months, although 80.6% (29/36) of preparations showing an RC *p* < 95%. Similarly, a statistically significant correlation between total activity of the preparation and RCP was identified for all preparations combined and for those from Generator 1 (Spearman correlation, *p* = 0.0011 and *p* < 0.001, respectively), whereas this association was not significant for Generator 2 (Pearson correlation, *p* = 0.35). Finally, five operators performed the studied preparations: three radiopharmacists (with 2–8 years of experience) and two radiopharmacy residents in training. Each operator performed between 20 and 24 preparations. Overall, one radiopharmacy resident achieved a significantly lower mean RCP (one-way ANOVA, *p* < 0.01). However, when RCP values obtained with Generator 1 and Generator 2 were analyzed separately, this difference was no longer statistically significant (one-way ANOVA, *p* = 0.46, and global Kruskal–Wallis test, *p* = 0.69, respectively).

**Table 2:** RCP values obtained with the two ^68^ Ge/ ^68^ Ga generators used during the study period

	Generator 1			Generator 2			Generators 1 + 2		
	Aliquot 1	Aliquot 2	Total	Aliquot 1	Aliquot 2	Total	Aliquot 1	Aliquot 2	Total
Number ( *n* )	29	29	58	18	18	36	47	47	94
Mean RCP ± SD (%)	96.87 ± 3.30	97.79 ± 1.74	97.33 ± 2.66	88.52 ± 5.81	92.53 ± 4.12	90.53 ± 5.36	93.67 ± 5.99	95.78 ± 3.84	94.72 ± 5.12

Abbreviations: RCP, radiochemical purity; SD, standard deviation.

In our center, the aliquoting of kits combined with radiolabeling using a single generator eluate replaced the use of a full kit for each preparation. Considering the initial cost of the generator and its amortization over its useful lifetime based on the number of elutions, the cost per elution was estimated at 475 €. The cost of the kit was 1250 €, while the minor consumables required for radiolabeling (tubing, needles, adapters) were considered negligible. Whether a complete kit or an aliquot is used, three patient doses are prepared per batch throughout the generator's lifetime. Without aliquoting, the cost per patient dose was 575 €; with aliquoting, it decreased to 367 €, representing a 36% cost reduction.

## Discussion

The widespread availability of generator-produced gallium-68 positioned PET imaging with ^68^ Ga-labeled tracers as a reliable approach to effectively facilitate the rapid transition of experimental radiopharmaceuticals into broader clinical use. 12 The kit-based formulation significantly simplifies the radiolabeling process and its implementation in routine clinical practice, as demonstrated with DOTA-functionalized somatostatin analogs. 10 13 In particular, the edotreotide kit SOMAKIT-TOC approved by the European Medicines Agency, has highlighted the advantages of this format in enhancing process robustness and standardizing the production of this radiotracer. 14 For nuclear medicine departments equipped with a single PET camera, and depending on the eluted activity from the ^68^ Ge/ ^68^ Ga generator, a single [ ^68^ Ga]Ga-edotreotide preparation typically enables two to four imaging procedures; potentially more, depending on the dosage set and acquisition methods. Given this limited number, we sought to maximize the number of examinations achievable from a single kit, using techniques inspired by previous studies conducted with PSMA-11 kits 15 16 and formulations for ^99m^ Tc radiolabeling. 17 18 19 20 21 Three original procedures are presented herein, based on the use of two GALLIAD generator eluates and/or the division of a single kit into two aliquots. As with the protocol described in the SmPC, these alternative methodologies must ensure minimal contamination with ionic metal impurities, particularly by using coated needles and sterile type I glass vials with bromobutyl rubber stoppers. 22 Such impurities have been shown to negatively impact the radiolabeling efficiency of somatostatin analogs with ^68^ Ga. 23 24 Also, the volume of buffer solution used in each protocol was adjusted according to the volume of HCl 0.1 M introduced, to maintain the reaction pH between 3.2 and 3.8, which is optimal for ^68^ Ga radiolabeling. 25 Overall, modifications to the original preparation method must ensure the sterility of the final products, which was confirmed in this study.

Among the three original procedures developed, all proved very easy to implement due to the minimal modifications applied to the protocol described in the SmPC. Quality control of the test radiolabeling triplicates showed excellent mean RCP by rTLC at EoS for all three procedures, supporting their suitability for clinical use. Lower values and a slight decrease in RCP over time was observed by rHPLC for all three original methods, suggesting that these radiopharmaceuticals should ideally be used within 1 hour after preparation. The lowest RCP values by rHPLC were observed with Procedure 4, likely due to the lower amount of DOTA-peptide combined with a high ^68^ Ga activity. The amount of peptide precursor is a well-known critical parameter in ^68^ Ga radiolabeling, and all previously reported developments of cold kits based on somatostatin analogs have used peptide amounts ranging from 35 to 50 µg, 26 27 28 compared with around 20 µg in the present aliquoting assays.

Procedure 3 was implemented in routine clinical practice at our center and enabled a substantial reduction in the operational costs associated with ^68^ Ga-PET imaging, as it allowed twice as many examinations to be performed from a single cold kit. More than 280 imaging procedures were performed using aliquoted preparations, without any equivocal imaging results. However, aliquoting logically appears to reduce the robustness of the cold kit formulation, as 33% of the preparations produced using Procedure 3 exhibited RCP by rTLC between 80 and 95%. Two preparations experienced critical failures, with RCP between 75 and 80%, and were not administered to patients. In cases where the RCP approaches the minimum threshold recommended in the SmPC, the question arises regarding the radiopharmacist responsibility in releasing the batch and the physician's approval for its clinical use, as low RCP could lead to imaging artifacts and reduced diagnostic accuracy. 29

In our institution, quality controls by rTLC are performed on every radiopharmaceutical preparation prior to clinical use. When the RCP falls below the expected minimum threshold, the decision to use the product off-label or not is jointly made by the radiopharmacist and the nuclear medicine physician. The RCP value is digitally recorded, and each patient dose is traceable to its corresponding preparation batch. The investigation of predictive factors for radiolabeling failure first highlighted a potential influence of the generator batch on RCP, which could possibly be explained by differences in conditioning quality or in the ^68^ Ge raw material between generators. 30 Interestingly, reported RCP values appeared to be operator-dependent, with lower results obtained by a radiopharmacy resident still in training. Due to the additional aliquoting step, Procedure 3 may require particular meticulousness and therefore more extensive operator training. A correlation between higher activity levels in the preparation and reduced RCP also seems to exist and has been observed in routine practice, although it contrasts with the potential negative impact of generator aging on RCP. These two parameters should therefore be further investigated over a longer period and across a greater number of generator batches to better characterize their actual relationship.

Further optimization of the protocols described herein may help reduce variability and ensure more consistent RCP. For example, precise aliquoting could be implemented, potentially by weighing the volumes of WFI added to and withdrawn from the kit during aliquoting, to ensure an exactly equal distribution between aliquot 1 and aliquot 2. Similarly, systematically incorporating a gentle mixing step (without inverting the vial, to avoid potential leaching of competing metals from the vial septum) between generator elution into the kit and the heating step could be beneficial to ensure proper mixing of the buffer and hydrochloric acid, as previously demonstrated with PSMA-11 kits. 31 Finally, additional comparative clinical validation would ideally be required to evaluate the diagnostic performance of radiopharmaceuticals prepared using these alternative protocols.

## Conclusion

In this study, we demonstrated that the SOMAKIT-TOC cold kit formulation allows for scalable radiolabeling procedures beyond its approved protocol. Aliquot-based and dual-generator strategies can be implemented with minimal technical adjustments and provide viable alternatives for increasing radiopharmaceutical yield or reducing operational costs. However, increased variability in RCP was observed with aliquoted preparations in clinical practice. These findings underscore the importance of careful quality control and risk assessment when deviating from approved protocols. Optimization of reaction parameters and further clinical validation are recommended before widespread adoption of these alternative methods in routine practice.
