# Supplementary material for: Scalable 68 Ga Cold Kit Radiolabeling: High-Throughput Preparation of [ 68 Ga]Ga-Edotreotide via Aliquoting and Dual Generator Strategy
Source: World J Nucl Med. 2026 Jan 5;25(1):19–27. doi: 10.1055/s-0045-1814733 (PMC13288594; doi:10.1055/s-0045-1814733)
Supplement: Supplementary file 1 — Supplementary Material [file 10-1055-s-0045-1814733-s2580010.pdf]

Details of radiochemical purity obtained for test batches with each procedure

Procedure 1 (conventional radiolabeling with 1 generator)											
Assay 1	RCP in rTLC	RCP in rHPLC	pH	Assay 2	RCP in rTLC	RCP in rHPLC	pH	Assay 3	RCP in rTLC	RCP in rHPLC	pH
t_0	99.45	91.59	3.60	t_0	99.05	94.15	3.60	t_0	98.06	93.66	3.60
t_{1h}	99.51	91.55	3.60	t_{1h}	98.76	93.06	3.60	t_{1h}	98.12	92.62	3.60
t_{2h}	99.18	91.25	3.60	t_{2h}	98.91	91.90	3.60	t_{2h}	98.01	91.69	3.60
t_{3h}	98.85	90.36	3.60	t_{3h}	96.99	91.06	3.60	t_{3h}	97.84	91.41	3.60
t_{4h}	98.12	90.56	3.60	t_{4h}	97.56	90.91	3.60	t_{4h}	97.96	90.43	3.60
Mean	99.02	91.06	3.60	Mean	98.25	92.22	3.60	Mean	98.00	91.96	3.60
%SD	0.57	0.57	0.00	%SD	0.92	1.38	0.00	%SD	0.11	1.23	0.00
**Activity at EoS:** 926 MBq				**Activity at EoS:** 896 MBq				**Activity at EoS:** 917 MBq			

Abbreviations: EoS, end of synthesis; RCP, radiochemical purity; rHPLC, radio-high performance liquid chromatography; rTLC, radio-thin layer chromatography; SD, SD, standard deviation.

Procedure 2 (radiolabeling with two generator eluates for one full kit vial)											
Assay 1	RCP in rTLC	RCP in rHPLC	pH	Assay 2	RCP in rTLC	RCP in rHPLC	pH	Assay 3	RCP in rTLC	RCP in rHPLC	pH
t_0	97.65	90.42	3.60	t_0	99.85	92.10	3.60	t_0	99.04	92.20	3.60
t_{1h}	98.22	88.00	3.60	t_{1h}	99.77	88.35	3.60	t_{1h}	99.40	90.45	3.60
t_{2h}	98.15	86.80	3.60	t_{2h}	99.71	87.13	3.60	t_{2h}	99.88	90.13	3.60
t_{3h}	98.09	85.31	3.60	t_{3h}	98.60	85.93	3.60	t_{3h}	95.31	89.69	3.60
t_{4h}	99.53	85.94	3.60	t_{4h}	99.80	84.94	3.60	t_{4h}	98.57	88.76	3.60
Mean	98.33	87.29	3.60	Mean	99.55	87.69	3.60	Mean	98.44	90.25	3.60
%SD	0.71	2.02	0.00	%SD	0.53	2.78	0.00	%SD	1.81	1.26	0.00
**Activity at EoS:** 1550 MBq				**Activity at EoS:** 1630 MBq				**Activity at EoS:** 1378 MBq			

Abbreviations: EoS, end of synthesis; RCP, radiochemical purity; rHPLC, radio-high performance liquid chromatography; rTLC, radio-thin layer chromatography; SC, SD, standard deviation.

Procedure 3, aliquot 1 (radiolabeling with one generator eluate for one aliquoted kit vial)											
Assay 1	RCP in rTLC	RCP in rHPLC	pH	Assay 2	RCP in rTLC	RCP in rHPLC	pH	Assay 3	RCP in rTLC	RCP in rHPLC	pH
t_0	98.93	90.26	3.60	t_0	99.53	88.20	3.60	t_0	98.34	94.90	3.60
t_{1h}	98.63	91.41	3.60	t_{1h}	99.10	87.83	3.60	t_{1h}	98.62	92.04	3.60
t_{2h}	98.71	90.34	3.60	t_{2h}	99.49	87.12	3.60	t_{2h}	98.17	90.61	3.60
t_{3h}	99.15	89.97	3.60	t_{3h}	98.91	85.73	3.60	t_{3h}	97.68	90.49	3.60
t_{4h}	98.20	88.40	3.60	t_{4h}	98.07	86.23	3.60	t_{4h}	97.50	89.04	3.60
Mean	98.72	90.08	3.60	Mean	99.02	87.02	3.60	Mean	98.06	91.42	3.60
%SD	0.36	1.08	0.00	%SD	0.59	1.04	0.00	%SD	0.46	2.22	0.00
**Activity at EoS:** 1009 MBq				**Activity at EoS:** 969 MBq				**Activity at EoS:** 936 MBq			

Abbreviations: EoS, end of synthesis; RCP, radiochemical purity; rHPLC, radio-high performance liquid chromatography; rTLC, radio-thin layer chromatography; SD, SD, standard deviation.

 © 2026. The Author(s).

Procedure 3, aliquot 2 (radiolabeling with one generator eluate for one aliquoted kit vial)											
Assay 1	RCP in rTLC	RCP in rHPLC	pH	Assay 2	RCP in rTLC	RCP in rHPLC	pH	Assay 3	RCP in rTLC	RCP in rHPLC	pH
t_0	97.06	93.12	3.60	t_0	98.64	90.87	3.60	t_0	97.19	88.44	3.60
t_{1h}	98.85	92.39	3.60	t_{1h}	98.47	89.70	3.60	t_{1h}	96.21	86.44	3.60
t_{2h}	97.92	91.78	3.60	t_{2h}	98.08	89.37	3.60	t_{2h}	95.84	86.79	3.60
t_{3h}	97.23	91.03	3.60	t_{3h}	97.38	89.30	3.60	t_{3h}	96.21	85.05	3.60
t_{4h}	99.31	90.74	3.60	t_{4h}	97.56	88.96	3.60	t_{4h}	96.06	89.03	3.60
Mean	98.07	91.81	3.60	Mean	98.03	89.64	3.60	Mean	96.30	87.15	3.60
%SD	0.99	0.98	0.00	%SD	0.55	0.74	0.00	%SD	0.52	1.60	0.00
**Activity at EoS:** 957 MBq				**Activity at EoS:** 920 MBq				**Activity at EoS:** 890 MBq			

Abbreviations: EoS, end of synthesis; RCP, radiochemical purity; rHPLC, radio-high performance liquid chromatography; rTLC, radio-thin layer chromatography; SD, standard deviation.

Procedure 4, aliquot 1 (radiolabeling with two generator eluates for one aliquoted kit vial)											
Assay 1	RCP in rTLC	RCP in rHPLC	pH	Assay 2	RCP in rTLC	RCP in rHPLC	pH	Assay 3	RCP in rTLC	RCP in rHPLC	pH
t_0	97.24	84.67	3.60	t_0	95.22	85.79	3.60	t_0	94.45	90.98	3.60
t_{1h}	96.88	82.85	3.60	t_{1h}	95.05	85.36	3.60	t_{1h}	97.61	88.71	3.60
t_{2h}	98.27	82.19	3.60	t_{2h}	96.59	83.91	3.60	t_{2h}	97.30	87.38	3.60
t_{3h}	91.49	80.58	3.60	t_{3h}	96.24	82.73	3.60	t_{3h}	98.34	86.94	3.60
t_{4h}	98.16	79.07	3.60	t_{4h}	97.20	82.87	3.60	t_{4h}	99.10	86.28	3.60
Mean	96.41	81.87	3.60	Mean	96.06	84.13	3.60	Mean	97.36	88.06	3.60
%SD	2.81	2.15	0.00	%SD	0.91	1.40	0.00	%SD	1.77	1.86	0.00
**Activity at EoS:** 1503 MBq				**Activity at EoS:** 1459 MBq				**Activity at EoS:** 1335 MBq			

Abbreviations: EoS, end of synthesis; RCP, radiochemical purity; rHPLC, radio-high performance liquid chromatography; rTLC, radio-thin layer chromatography; SD, standard deviation.

Procedure 4, aliquot 2 (radiolabeling with two generator eluates for one aliquoted kit vial)											
Assay 1	RCP in rTLC	RCP in rHPLC	pH	Assay 2	RCP in rTLC	RCP in rHPLC	pH	Assay 3	RCP in rTLC	RCP in rHPLC	pH
t_0	93.28	86.16	3.60	t_0	98.52	91.20	3.60	t_0	99.70	91.45	3.60
t_{1h}	93.28	84.62	3.60	t_{1h}	98.77	90.27	3.60	t_{1h}	99.52	89.92	3.60
t_{2h}	92.98	83.83	3.60	t_{2h}	98.25	88.94	3.60	t_{2h}	99.75	88.47	3.60
t_{3h}	94.73	84.19	3.60	t_{3h}	98.87	87.57	3.60	t_{3h}	99.58	87.87	3.60
t_{4h}	93.45	84.60	3.60	t_{4h}	99.06	87.90	3.60	t_{4h}	98.80	87.53	3.60
Mean	93.54	84.68	3.60	Mean	98.69	89.18	3.60	Mean	99.47	89.05	3.60
%SD	0.68	0.89	0.00	%SD	0.32	1.55	0.00	%SD	0.39	1.62	0.00
**Activity at EoS:** 1390 MBq				**Activity at EoS:** 1380 MBq				**Activity at EoS:** 1369 MBq			

Abbreviations: EoS, end of synthesis; RCP, radiochemical purity; rHPLC, radio-high performance liquid chromatography; rTLC, radio-thin layer chromatography; SD, standard deviation.

 © 2026. The Author(s).

Representative spectra for [^{68}Ga]Ga-edotreotide obtained with each procedure

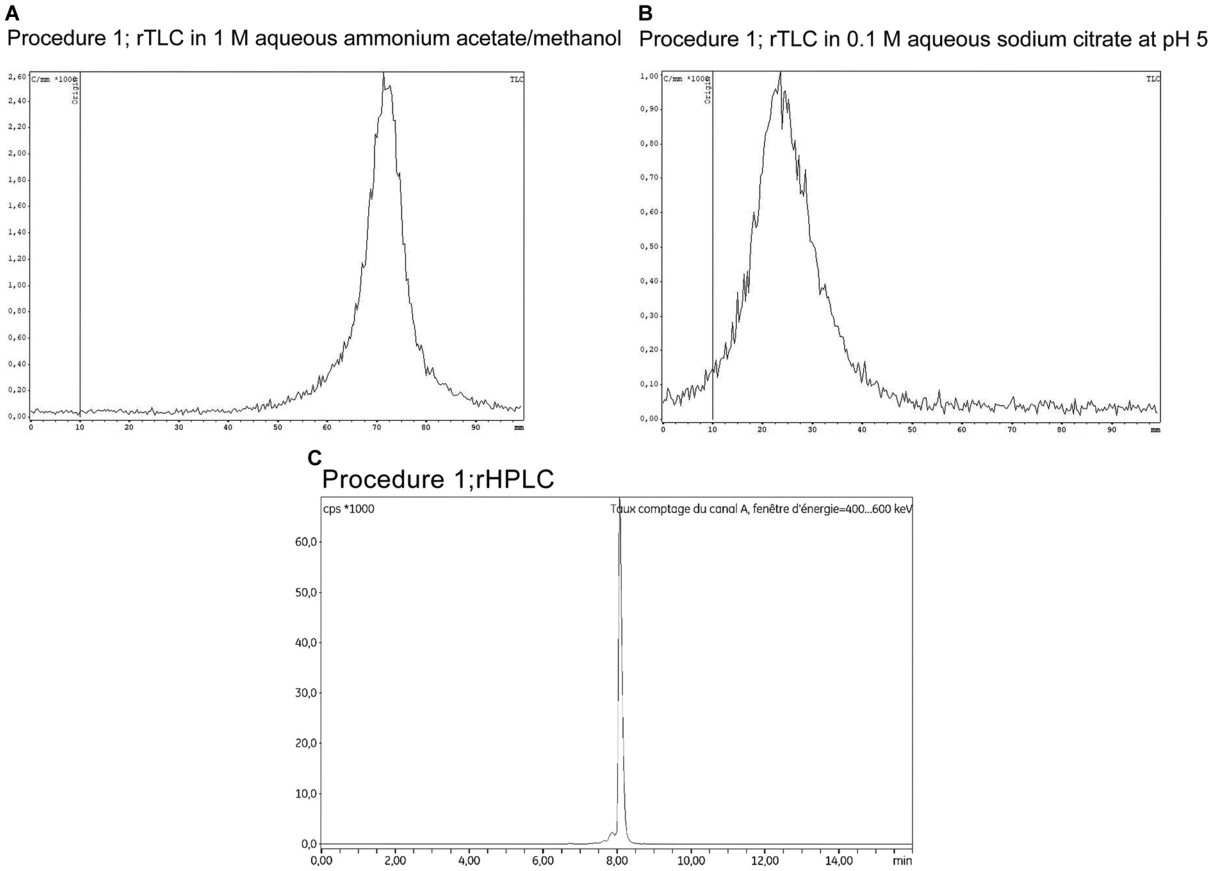

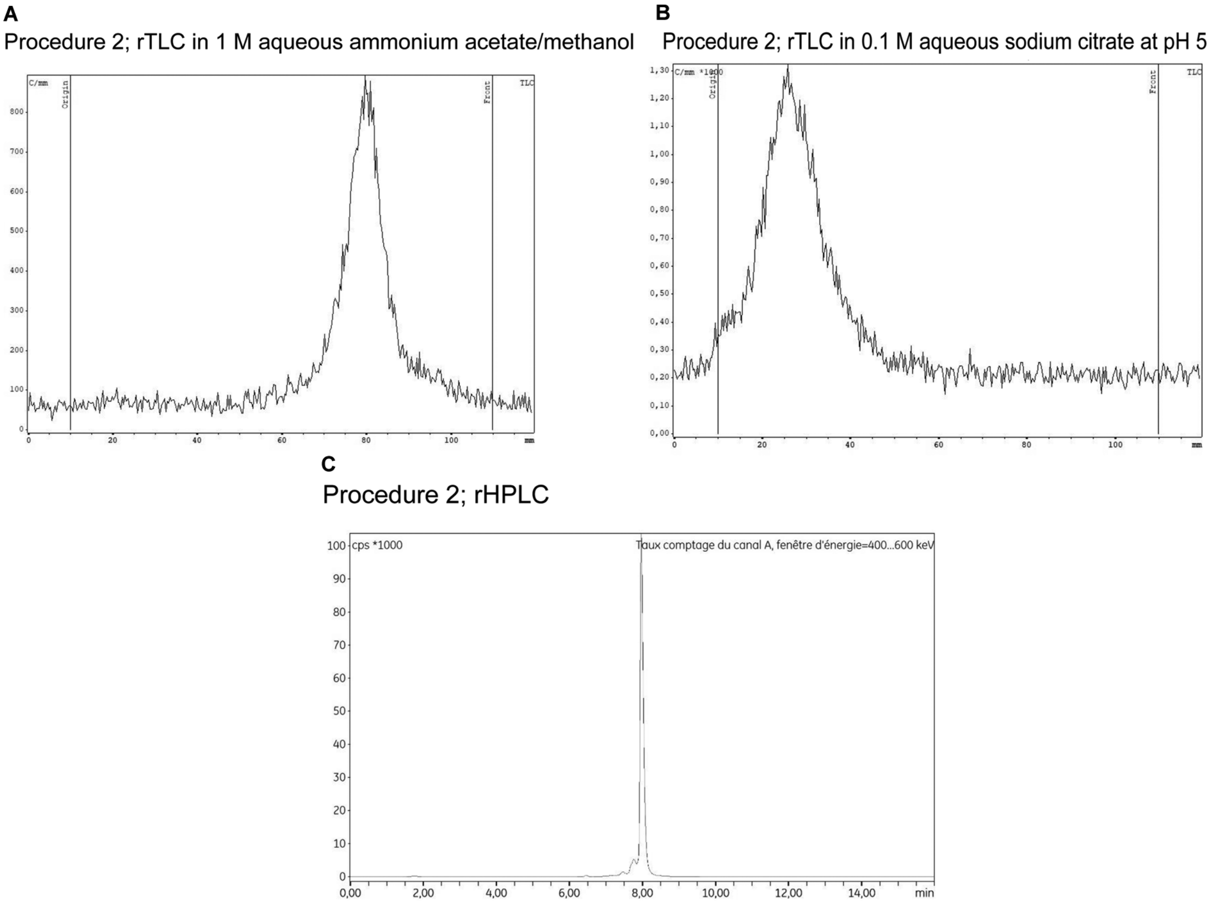

 © 2026. The Author(s).

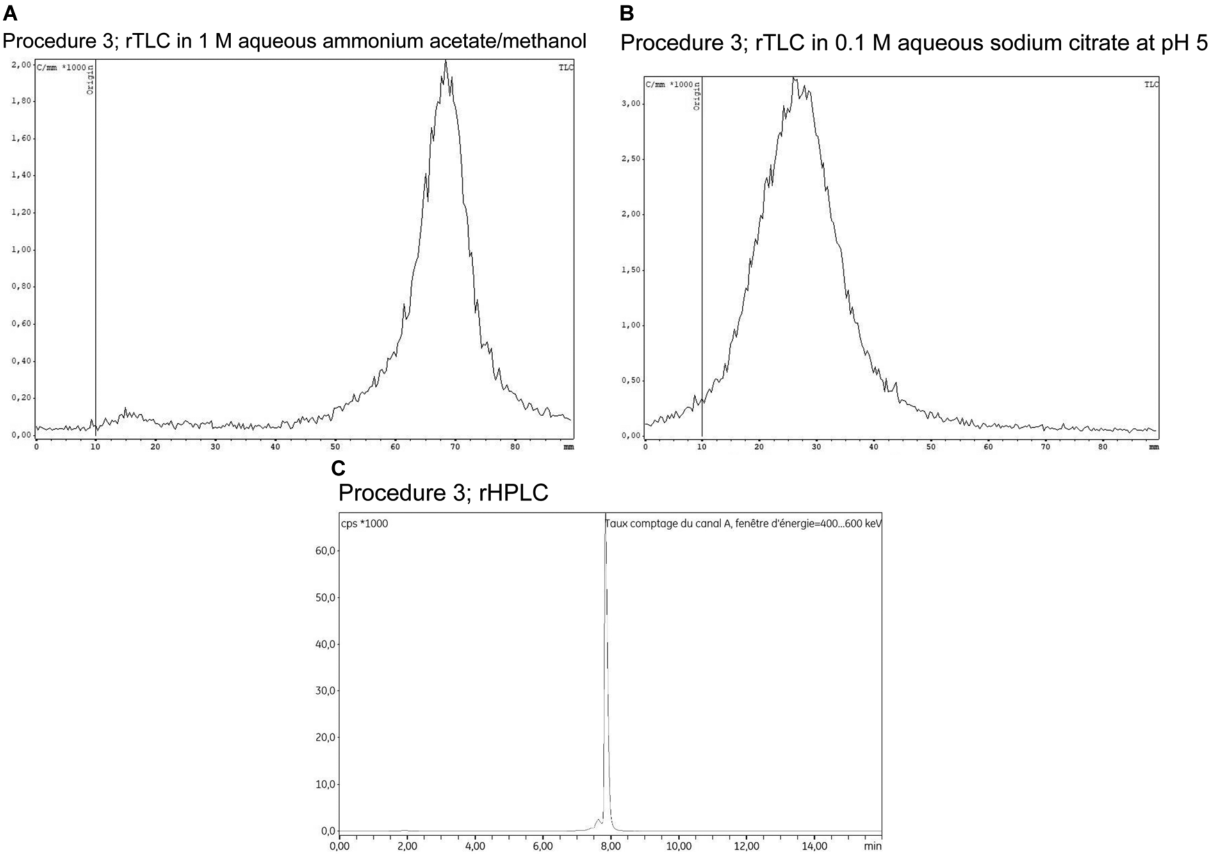
A
Procedure 3; rTLC in 1 M aqueous ammonium acetate/methanol
C/mm *1000
Origin
TLC
mm
B
Procedure 3; rTLC in 0.1 M aqueous sodium citrate at pH 5
C/mm *1000
Origin
TLC
mm
C
Procedure 3; rHPLC
cps *1000
Taux comptage du canal A, fenêtre d'énergie=400...600 keV
min

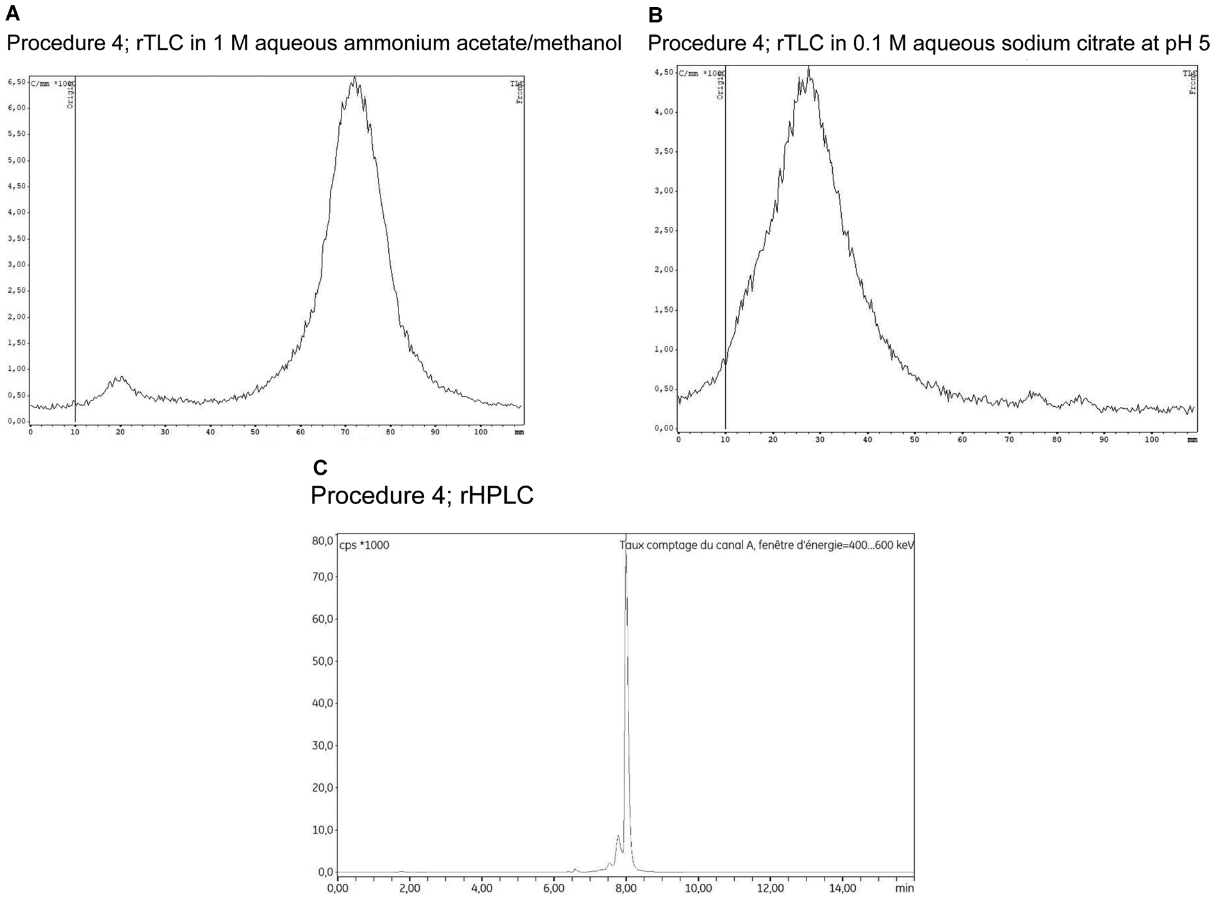
A
Procedure 4; rTLC in 1 M aqueous ammonium acetate/methanol
C/mm *1000
Origin
mm
B
Procedure 4; rTLC in 0.1 M aqueous sodium citrate at pH 5
C/mm *1000
Origin
mm
C
Procedure 4; rHPLC
cps *1000
Taux comptage du canal A, fenêtre d'énergie=400...600 keV
min

Representative spectra from the stability assessment of edotreotide in solution

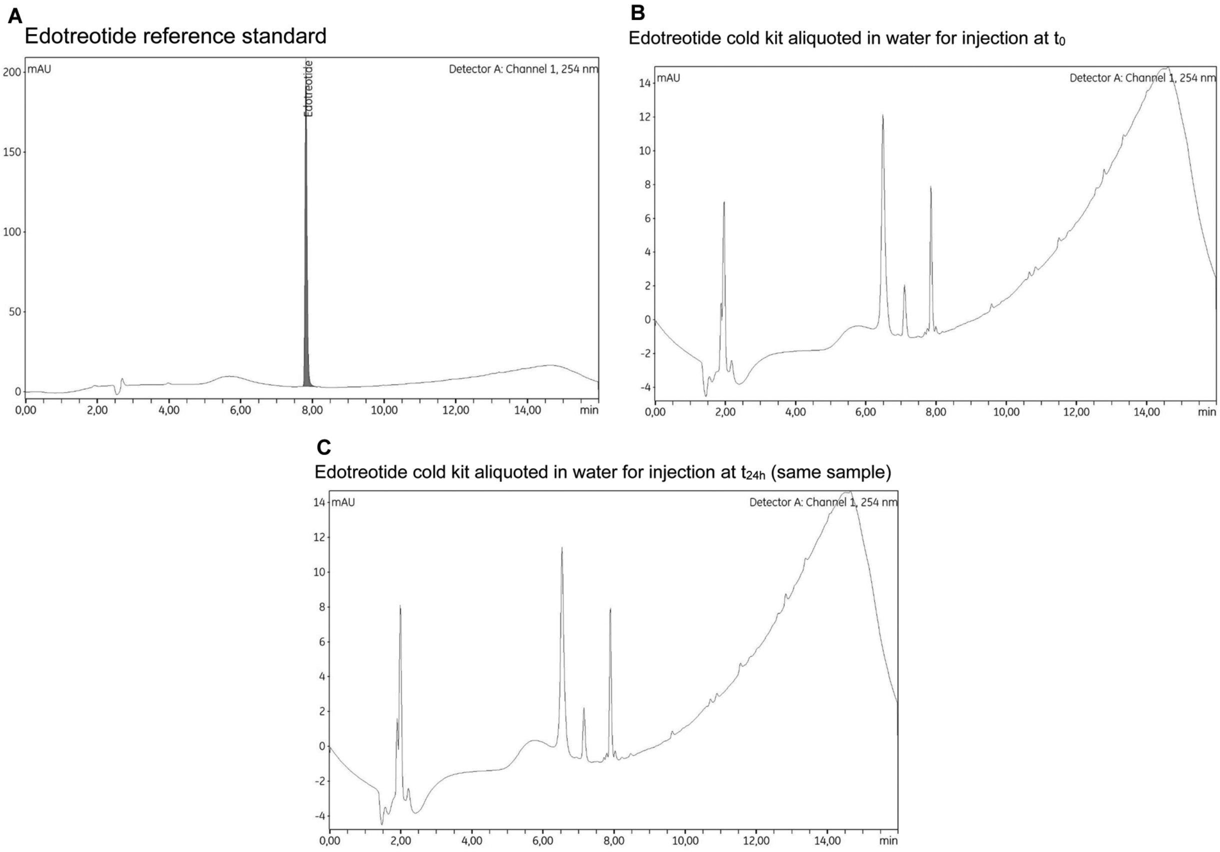

Details of radiochemical purity obtained in real life

Overall data for procedure 1 and procedure 3

	Procedure 1	Procedure 3, aliquot 1	Procedure 3, aliquot 2
Number of radiolabelings	166	48	48
Mean RCP in rTLC (%)	97.32 ± 1.80	93.67 ± 5.99	95.78 ± 3.84
Minimum RCP in rTLC (%)	86.45	76.88	83.83
Maximum RCP in rTLC (%)	99.56	99.53	99.92
Median RCP in rTLC (%)	97.70	96.40	97.21
Mean $^{68}Ga^{3+}$ (%)	0.70 ± 0.95	0.97 ± 1.80	0.84 ± 1.42
Mean ^{68}Ga colloids (%)	1.97 ± 1.25	5.36 ± 5.25	3.38 ± 3.15
Non-compliant $^{68}Ga^{3+}$ content (*n* [%])	9 (5.42)	6 (12.50)	7 (14.58)
Non-compliant ^{68}Ga colloids content (*n* [%])	17 (10.24)	23 (47.92)	18 (37.50)
Non-compliant overall RCP (*n* [%])	10 (6.02)	18 (37.50)	15 (31.25)

Abbreviations: EoS, end of synthesis; RCP, radiochemical purity; rTLC, radio-thin layer chromatography.

Detailed results for procedure 3

Blue: generator 1 Green: generator 2	$^{68}Ga^{3+}$ impurities aliquot 1 (%)	^{68}Ga colloids aliquot 1 (%)	RCP aliquot 1 (%)	Activity at EoS aliquot 1 (MBq)	$^{68}Ga^{3+}$ impurities aliquot 2 (%)	^{68}Ga colloids aliquot 2 (%)	RCP aliquot 2 (%)	Activity at EoS aliquot 2 (MBq)	Time post-calibration of the generator (d)
06/06/2025	0.18	6.08	93.74	746	5.39	2	92.61	712	157
30/05/2025	2.35	8.49	89.16	741	3.53	11.56	84.91	709	150
23/05/2025	0.58	6.63	92.79	757	5.04	4.6	90.36	741	143
09/05/2025	0.56	4.23	95.21	751	2.15	14.02	83.83	691	129
25/04/2025	0.37	10.54	89.09	819	0.33	4.25	95.42	775	115
11/04/2025	1.05	6.11	92.84	858	3.93	2.14	93.93	795	101
04/04/2025	5.78	17.34	76.88	839	1.71	4.82	93.47	858	94
28/03/2025	0.28	3.17	96.55	893	0.09	2.09	97.82	868	87
21/03/2025	0.1	8.23	91.67	879	1.54	2.8	95.66	874	80
14/03/2025	0.17	17.86	81.97	915	0.45	5.59	93.96	913	73
07/03/2025	1.71	8.31	89.98	954	0.12	5.62	94.26	895	66
21/02/2025	0.68	20.58	78.74	1017	0.13	4.24	95.63	965	52
14/02/2025	0.04	4.99	94.97	915	0	3.19	96.81	913	45
07/02/2025	0.15	10	89.85	954	0.01	6.72	93.27	895	38
31/01/2025	0.02	13.78	86.2	1069	0.38	4.66	94.96	1009	31
24/01/2025	3.58	12.4	84.02	1088	2.44	3.31	94.25	1012	24
17/01/2025	2.56	9.56	87.88	1089	4.77	8.81	86.42	1001	17
10/01/2025	0.11	18.05	81.84	1083	0.1	11.92	87.98	1064	10
03/01/2025	1.53	3.07	95.4	465	0.22	2.62	97.16	454	335
27/12/2024	0.69	2.22	97.09	465	0.06	1.67	98.27	451	328
20/12/2024	4.81	2.59	92.6	477	0.15	6.91	92.94	477	321
13/12/2024	0.03	2.59	97.38	497	0.27	1.81	97.92	481	314
11/12/2024	0.48	0.99	98.53	512	1.21	1.37	97.42	496	312
06/12/2024	0.51	4.47	95.02	508	0.06	2.3	97.64	477	307
29/11/2024	0.57	2.96	96.47	520	0.36	0.98	98.66	491	300
27/11/2024	0.27	2.25	97.48	493	0.05	1.04	98.91	506	298
15/11/2024	0.01	0.84	99.15	536	0.36	6.85	92.79	512	286
08/11/2024	0.06	2.49	97.45	543	0.2	1.87	97.93	540	279
25/10/2024	0.42	2.64	96.94	567	0.13	4.42	95.45	561	265
18/10/2024	1.61	0.87	97.52	574	0.1	1.6	98.3	560	258
11/10/2024	9.87	8.41	81.72	616	0.33	1.32	98.35	571	251
04/10/2024	0.1	1.39	98.51	578	0.15	3.13	96.72	570	244
18/09/2024	0.26	2.25	97.49	617	1.05	2.78	96.17	594	228
13/09/2024	0.3	2.81	96.89	635	0.39	2.38	97.23	609	223
06/09/2024	0.28	2.13	97.59	638	0.12	0.33	99.55	612	216
23/08/2024	0.04	0.94	99.02	641	0.66	0.63	98.71	630	202
16/08/2024	0.45	4.26	95.29	699	0.05	2.74	97.21	673	195
09/08/2024	0.22	0.61	99.17	691	0.13	0.58	99.29	674	188
19/07/2024	0.15	1.62	98.23	723	0.34	0.77	98.89	685	167
12/07/2024	0.94	2.66	96.4	753	0.41	0.8	98.79	713	160
05/07/2024	0.09	1.36	98.55	763	0.03	0.13	99.84	759	153

(*Continued*)

 © 2026. The Author(s).

(*Continued*)

Blue: generator 1 Green: generator 2	$^{68}Ga^{3+}$ impurities aliquot 1 (%)	^{68}Ga colloids aliquot 1 (%)	RCP aliquot 1 (%)	Activity at EoS aliquot 1 (MBq)	$^{68}Ga^{3+}$ impurities aliquot 2 (%)	^{68}Ga colloids aliquot 2 (%)	RCP aliquot 2 (%)	Activity at EoS aliquot 2 (MBq)	Time post-calibration of the generator (d)
14/06/2024	0.09	1.37	98.54	815	0	1.94	98.06	800	132
07/06/2024	0.25	1.85	97.9	833	0.22	0.43	99.35	812	125
06/06/2024	0.03	0.57	99.4	827	0.18	1.85	97.97	819	124
16/05/2024	0.15	1.51	98.34	890	0.04	2.73	97.23	875	103
02/05/2024	0.68	3.81	95.51	875	0.17	0.45	99.38	938	89
22/03/2024	0.43	0.04	99.53	1039	0	0.08	99.92	989	48

Abbreviations: EoS, end of synthesis; RCP, radiochemical purity.

 © 2026. The Author(s).
